# A novel approach to exploring infant gaze patterns with AI-manipulated videos

**DOI:** 10.1038/s41598-025-02727-z

**Published:** 2025-06-20

**Authors:** Charlotte Viktorsson, Tobias Lundman, Kim Astor

**Affiliations:** 1 Development and Neurodiversity Lab, Department of Psychology, Uppsala University, Uppsala, Sweden; 2 Uppsala Child and Baby Lab, Department of Psychology, Uppsala University, Uppsala, Sweden

**Keywords:** Eye tracking, Infants, AI, Eye-mouth-index, Gaze following, Social attention, Human behaviour, Behavioural methods

## Abstract

Eye tracking is a widely used tool to study infant development, but creating diverse stimuli while maintaining high control over confounding variables can be challenging. In this proof-of-concept study, we examined an innovative way to generate ecologically valid stimuli using AI technology, in order to create videos that can be used in culturally diverse settings. Using the eye-mouth-index (EMI), a commonly used paradigm in infant eye tracking, we examined the consistency of eye tracking measures across original videos and two types of AI-manipulated videos in a sample of 46 infants aged 12–14 months. We found a very strong correlation of the EMI across original and AI videos (*r* = 0.873–0.874), and there were no statistically significant differences between mean EMI in the original and AI conditions. Additionally, we created culturally diverse videos to measure gaze following, and found that children followed the gaze of the people in the AI-manipulated videos in an expected manner. In conclusion, AI technology provides promising tools to create ecologically valid and culturally diverse stimuli, that can be used to conduct studies in a wide range of settings and to examine the generalizability of earlier findings in the field of developmental psychology.

**Supplementary Information:**

The online version contains supplementary material available at 10.1038/s41598-025-02727-z.

## Introduction

Eye-tracking has revolutionized our understanding of early development by allowing us to assess how pre-verbal infants perceive and engage with the world through eye gaze, one of their earliest forms of interaction. By providing critical insights into infant cognition, this methodology forms the foundation for broader theories on human development^10,30^. However, a significant concern arises from the overwhelming dominance of samples drawn from White infants in Western Europe and North America^14,29^, reflecting a well-known issue in psychological research at large^6,19^. This lack of diversity risks skewing our understanding of development and raises questions about the global generalizability of existing findings and, in extension, the validity of existing theoretical frameworks.

The need for global expansion of research brings with it a need for stimuli that can be used across different cultures and settings, which may be a difficult task. Efforts to replicate findings in majority world contexts demonstrate the role of early experience-dependent development: Already at 3 months of age, infants prefer to look at faces of their own race over faces of other races^21^, and by 9–10 months, they differentiate faces of their own race better than faces of other races^31^. This phenomenon, known as perceptual narrowing, highlights the potential for confounding factors when using stimuli that are not culturally or contextually appropriate. Typically, this issue is addressed by filming equivalent videos in the relevant cultural setting, a process that demands additional time and resources. Moreover, even if the actors from different geographical backgrounds follow the same instructions, subtle variations in intonation, facial expressions, and posture can still arise. Additionally, replicating a consistent filming environment across settings can present challenges. These factors make creating diverse, high-quality stimuli a complex and often daunting task, with the final result sometimes falling short of expectations. A potential solution for creating stimuli with identical content but varying appearances is generative video editing powered by artificial intelligence (AI). If the appearance of an actor in an original video stimulus can be manipulated to resemble a different individual while keeping the exact timing and content, it provides an opportunity to quickly, easily, and cost-effectively create diverse stimuli suitable for use across and within cultural contexts.

In this proof-of-concept study, we explore the feasibility of creating and using AI-manipulated stimuli in infant research, in relation to two well-established paradigms: the eye-mouth-index (EMI) and gaze following (GF). The *eye-mouth-index* refers to the proportion of looking time at the eyes versus total looking time at both eyes and mouth. Eye and mouth looking have been studied throughout infancy and toddlerhood (e.g.,^11,23,32^), and is linked to language development^26,32^. The reason for including the EMI in this study is that examining the generalizability of the EMI requires diverse stimuli that minimize unnecessary confounders, and AI-manipulated stimuli offer a promising solution to address this need. In addition, this measure takes both eye and mouth looking into account, and it has been found that infants show large individual differences in EMI patterns, that are primarily explained by genetic factors^32^. Therefore, instead of examining a general face or eye bias, we can study individual gaze patterns related to important features of the face.

*Gaze following* is the ability to synchronize visual attention with others towards external objects. Typically, it is studied by presenting a video where an adult is centered between two toys. The adult then looks at one of the toys, and the primary measure is whether the child follows that gaze and looks at the same toy^2^. The tendency to follow the gaze of adults enables infants to reduce the complexity of their attentional choices and directs the focus towards the most relevant information for their learning and environment. This ability is central to attention sharing and social cognition^16,25^ and has been closely linked to language development (see^9^) for a review). While GF has been studied using a range of different stimuli (even including inanimate objects, e.g.^12,24^, few cross-cultural comparisons have been conducted, and those that exist often involve notable differences in the stimuli (e.g.,^4^). Variation in perceptual saliency, spatial layout, ostensive cues, facial expressions, and infant arousal significantly influences infant GF (e.g.,^4,8,10,13,15,20^). This likely explains why studies from the same lab with similar demographics report substantial differences within the same age group (cf.,^1,3^), and raises potential concern for consistency across diverse stimuli. AI-manipulated videos offer a promising tool for creating diverse stimuli tailored to different cultural contexts while maintaining identical underlying behaviors. While the EMI was included in order to create a suitable comparison between original and AI material, GF stimuli were included to show the extent of cultural and gender diversity that can be produced in more complex scenes.

To date, no study has yet explored the feasibility of creating AI-videos to be used in infant research, or examined whether infants exhibit the same gaze pattern when viewing AI-manipulated videos as compared to non-AI original videos. This question is crucial to answer before AI-manipulated stimuli can be reliably integrated into infant research as a viable alternative to recordings of human actors. Here, in a sample of 12–14-month-olds, we investigated whether infants look differently at AI-manipulated videos than the original videos, focusing on the EMI and GF paradigms. At this developmental age, social attention is well-established and infants are sensitive to the uncanny valley^22^, meaning that if infants at this age do not respond differently to AI-generated content, it is unlikely that younger infants would. As this is the first study examining differences in responses between real and AI-generated stimuli in infants, our hypotheses were necessarily exploratory. However, due to carefully matched visual and behavioral features of our stimuli, we expected no systematic differences. While infants can be sensitive to the appearance of human actors^17,21^, they also often respond in similar ways to a range of agents, including inanimate or simplified ones^12,24^. Thus, if the AI-manipulated videos preserve key social cues and appear naturalistic to infants, we reasoned that they would engage with them as they would with the original videos. This pragmatic, appearance-based rationale led us to predict no difference in the EMI between AI-manipulated and original videos of women singing nursery rhymes (*H1*), and that infants would follow the actor’s gaze in the AI condition in a manner consistent with typical gaze-following behavior (*H2*).

## Methods

### Participants

Participants were recruited at the Child and Babylab at Uppsala University, from a list of families who had previously expressed interest in participating in research with their child. The families were contacted via email or by phone and then invited to the lab for the eye tracking session. Infants were included only if their age was 12–14 months (+/- 2 weeks), they had no uncorrected visual or hearing impairment, they were born at week 36 or later, and they had no traumatic brain injury or neurological condition. The visit lasted approximately 20–30 min in total. After the eye tracking session, the caregiver filled in a questionnaire on demographic information. The study was approved by the regional ethics board in Stockholm and was conducted in accordance with the Declaration of Helsinki. Written informed consent was obtained from all caregivers.

In total, data was collected from 55 children. Five were subsequently excluded due to technical issues, and in the EMI condition, another four were excluded due to an insufficient number of valid trials (see section Eye tracking measures). There were no significant differences between the included and the excluded children regarding age (t(53) = − 0.425, *p* = 0.673), family income (t(46) = 0.278, *p* = 0.782), or parental education level (t(48) = 0.279, *p* = 0.782). The final sample consisted of 46 children in the EMI condition and 50 children in the GF condition. As our primary analyses are related to the EMI, we report demographic statistics based on the sample included in those analyses (Table 1). While information on ethnicity was not collected, the recruitment and all test sessions were completed in Swedish, meaning that all infants had at least one caregiver fluent in Swedish.

**Table 1:** Demographic information (*N* = 46).

	Mean (SD)^a^ [Min; Max]
N females (%)	18 (39.1%)
Age (in days) at assessment	397.33 (24.65) [354; 439]
Family income^b^	7.63 (2.04) [1; 10]
Parental education level^c^	2.97 (0.41) [2; 4]

^a^Except for N females, which shows the frequency

^b^Family income per month. Scale 1–10 where 1 = < 20 K, 2 = 20–29 K, 3 = 30–39 K, 4 = 40–49 K, 5 = 50–59 K, 6 = 60–69 K, 7 = 70–79 K, 8 = 80–89 K, 9 = 90–99 K and 10 = > 100 K (SEK)

^c^Education level, averaged for both caregivers, on a scale from 1 to 4, where 1 = Primary, 2 = Secondary, 3 = Bachelor’s degree/Master’s degree/Higher vocational education, and 4 = Licentiate/doctoral degree

### Stimuli

**Fig. 1:**
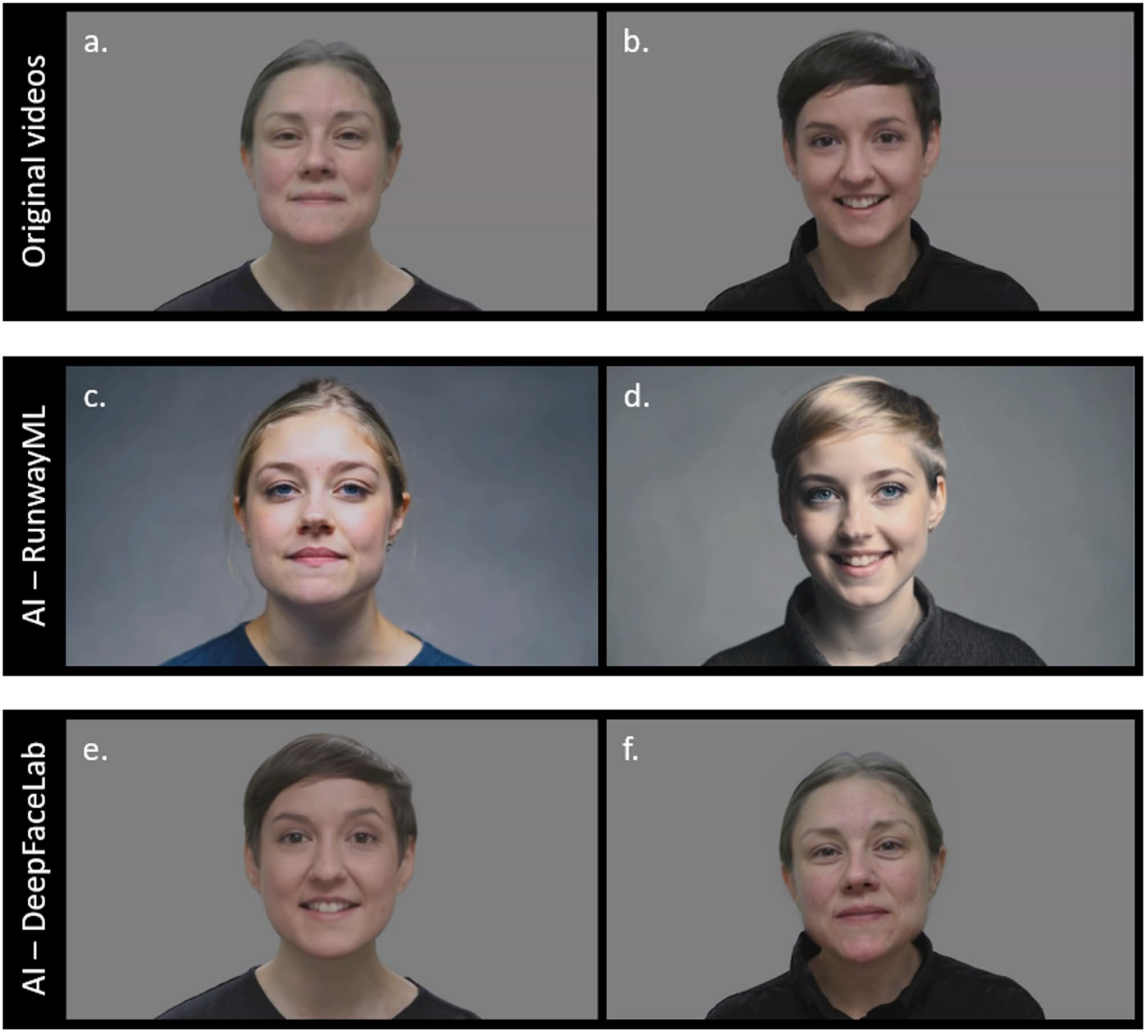
Frames from the videos presented in the EMI conditions. Original videos are shown on top, depicting person A (**a**) and person B (**b**); AI-manipulated versions utilizing RunwayML are shown in the middle (**c**, **d**), and AI-manipulated versions utilizing DeepFaceLab are shown on the bottom (**e**, **f**). Videos **e** and **f** were generated by combining elements from videos **a** and **b**. In the case of **e**, the face from video **b** was transferred onto the person in video **a**. In the case of **f**, the face from video **a** was transferred onto the person in video **b**. Despite these modifications, the sound and body movements in the AI-manipulated videos (**c**–**f**) remained unchanged from the original target videos (**a**, **b**). As a result, the audio and body movements are identical across all videos within each column (left and right, respectively). Both actors have provided written informed consent to publish identifying images and videos of them, and to have their videos/images manipulated using artificial intelligence.

The EMI stimuli consisted of two videos (Fig. 1a, b) that have been previously used in studies of infant gaze behavior (e.g.,^32^). In each video, a woman sings a common Swedish nursery rhyme. Based on the original videos, we generated new videos using two different AI tools, RunwayML and DeepFaceLab.

Two videos were created using RunwayML Gen-3 Alpha online software (https://runwayml.com/, see Fig. 1c, d). To generate a video, text prompts, images, and videos can be used as input. We used a combination of text prompts and video. See Supplementary Material S1 for the text prompts used in this study. The purpose was to make two AI-manipulated videos that could be validated against the original recordings. To ensure comparability and minimize confounding factors that may influence children’s gaze behavior, the AI-manipulated videos were designed to closely resemble actor performance in the original recordings while still exhibiting some differences in appearances (such as different hair and eye colors).

**Fig. 2:**
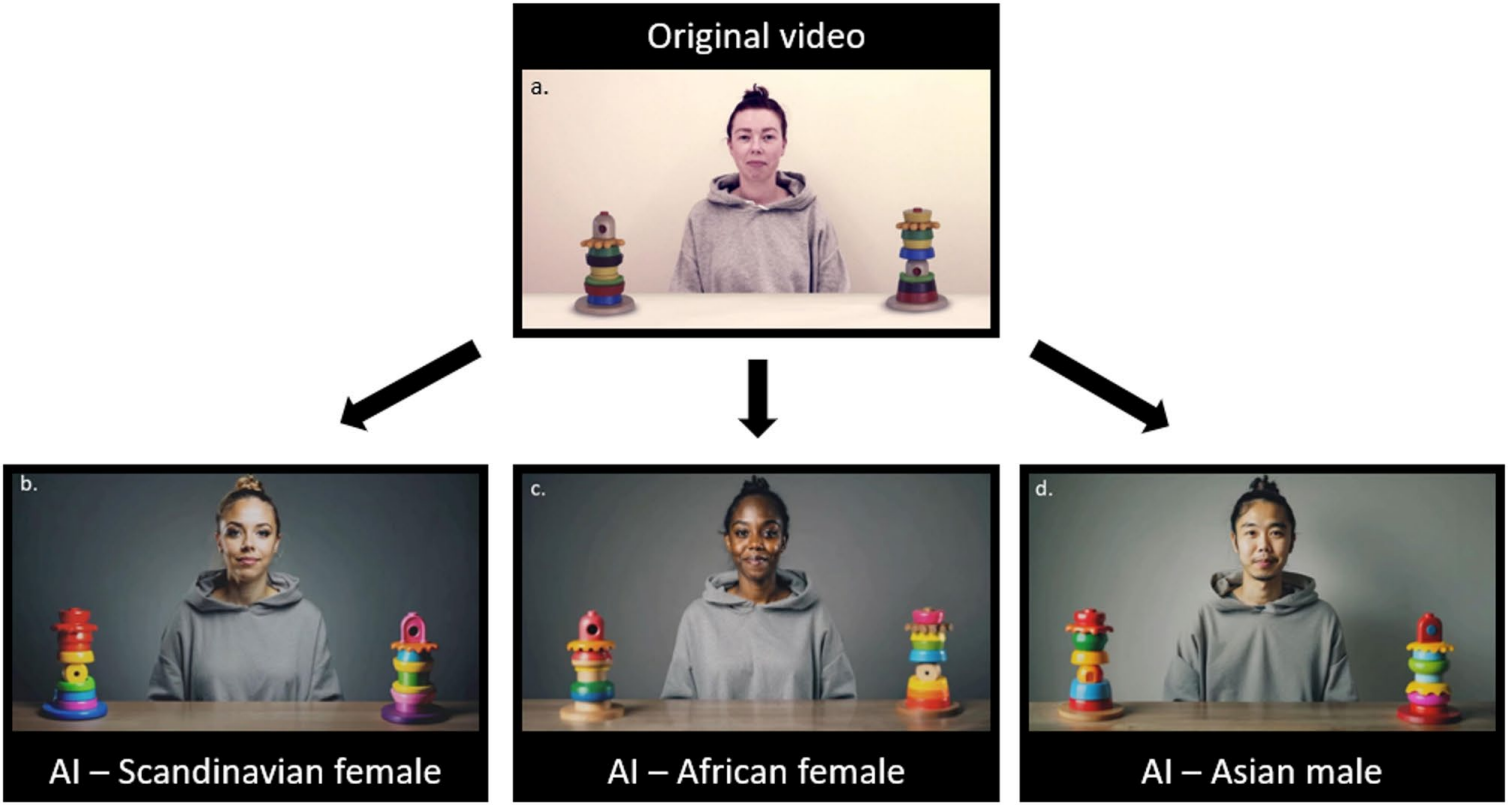
Frames from the videos presented in the GF conditions. Top left video (**a**) is the original, while the rest are AI-manipulated (**b**–**d**). The actor has provided written informed consent to publish identifying images and videos of them, and to have their videos/images manipulated using artificial intelligence.

In addition, two videos were created using DeepFaceLab, a software based on deep learning techniques to perform realistic face-swapping in videos (Fig. 2). These videos (Fig. 1e, f) were shown to 31 participants out of the total sample. The stimuli were included in order to explore gaze towards AI-manipulated material that is more similar to the original videos than the stimuli created with RunwayML. All details about the creation of these videos can be found in Supplementary Material S2.

The AI videos contained the same audio as the original videos, and because the AI-manipulated videos were slightly shorter than the original videos (due to restraints in the AI software), the first 10 s were analyzed for all videos (original and AI-manipulated).

The GF stimuli consisted of AI-manipulated videos (Fig. 2b–d) created based on one original video (Fig. 2a), that have been used in previous studies of GF (e.g.,^18^). Two versions were created of each video, one where the person is looking to the left and one where they are looking to the right. In total, the participants were shown six GF videos (the original video was not included in the stimuli shown in the eye tracking session). These videos were also created using the RunwayML Gen-3 Alpha online software (see Supplementary Material S1 for the text prompts used). These videos were created in order to display the large variation of possible generations that can be made based on one single original video.

### Eye tracking measures

Gaze data was collected using a Tobii TX300 eye tracker (120 Hz) integrated with a standard screen (24″). The infant was seated in their parent’s lap, approximately 60 cm from the screen. The videos included in this study were interspersed with other videos depicting social stimuli. Two versions of the experiment were created (version A and B), where the presentation of videos in version B was the opposite order as the presentation in version A. In both the EMI and GF condition, 56% were shown version A, while 44% were shown version B.

**Fig. 3:**
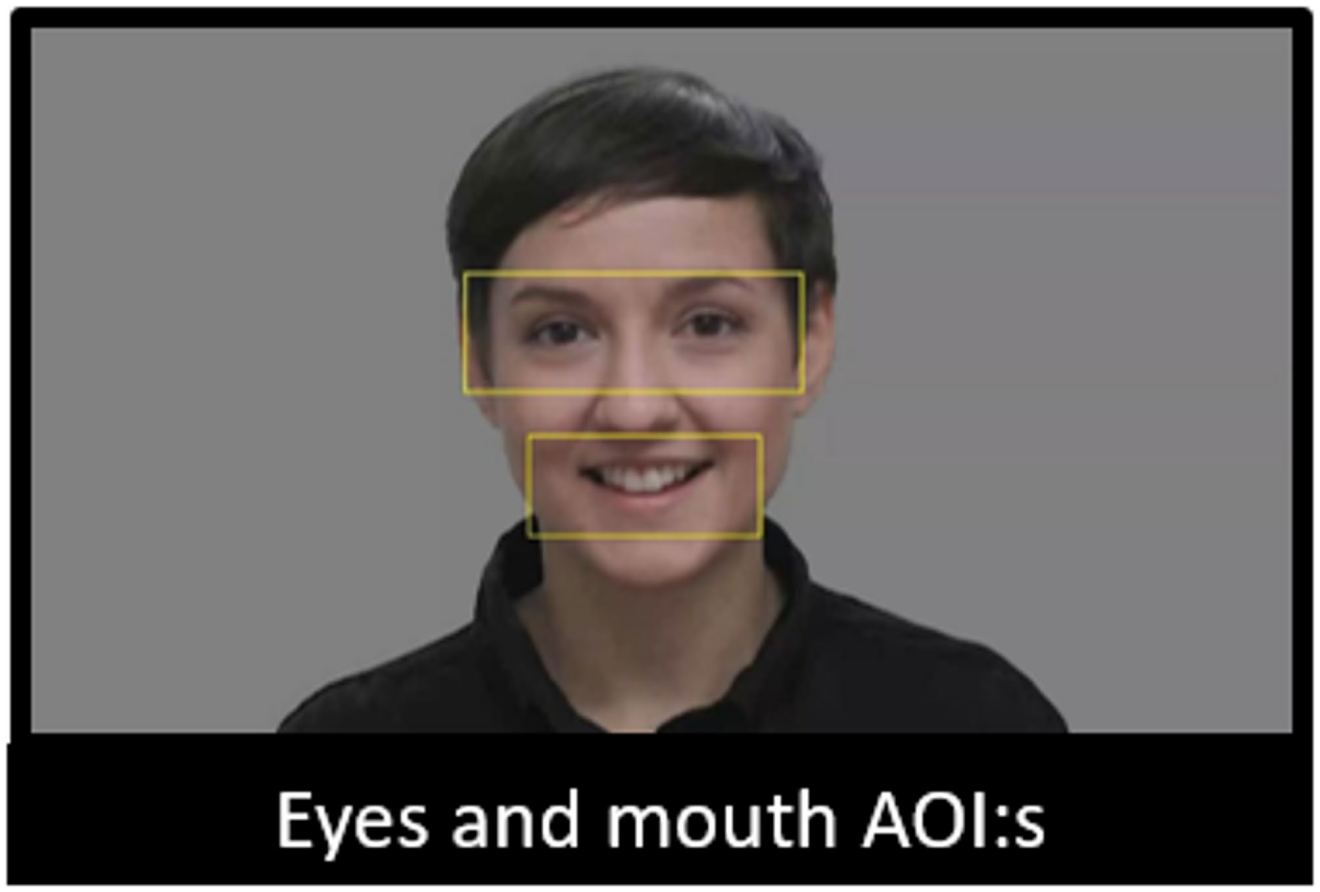
A frame from one of the original videos, depicting AOIs of eyes and mouth in the EMI condition

In the EMI conditions, all areas of interest (AOIs) were created by first analyzing the stimuli videos with the software OpenFace^5^, which detects faces via computer vision and extracts facial landmarks. We then used the x- and y-coordinates in pixels for different facial landmarks to create dynamic AOIs for each frame of the videos. The eye AOI was 510 × 180 pixels, and the mouth AOI was 350 × 150 pixels (see Fig. 3). The EMI was calculated based on the total looking time at the eyes divided by the total looking time at both eyes and mouth. In addition, we created a face AOI with a horizontal radius of 300 pixels and a vertical radius of 400 pixels.

Due to the time-consuming process of creating the DeepFaceLab videos, the first 15 participants were shown two original videos of person A, two original videos of person B, and one Runway-ML AI video of each person. To keep the total duration of the experiment the same across all participants, the remaining participants were shown one original video of each person and two AI videos of each person (made with RunwayML and DeepFaceLab, respectively). The reason for not including more videos of each person was the fact that these videos were interspersed with other social videos (related to other research projects), and we wanted the eye tracking session to be short enough for the children to keep their focus on the stimuli.

A trial was classified as invalid if the participant looked at the face for less than 2.5 s of the total trial duration. In order to be included in further analyses, a participant needed to have at least one valid trial for an original video of both person A and person B, and one valid trial for an AI video of each person. In total, four participants were excluded due to these criteria.

The EMI score was highly correlated across original videos with person A and person B (*r* = 0.79), we therefore combined the score for videos depicting person A and B, for both the original videos and the AI videos.

**Fig. 4:**
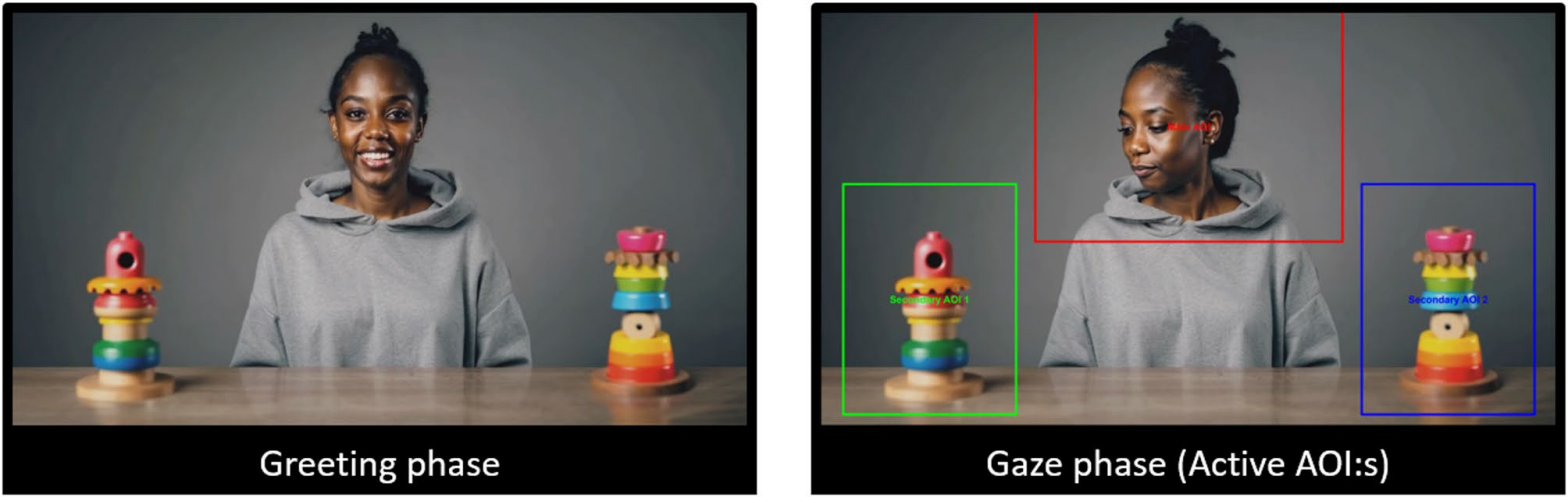
A frame from one of the videos in the AI-manipulated GF task depicting the greeting phase (left) and gaze phase with AOIs (right)

In the GF condition, three rectangular AOIs were created: one covering the actor (800 × 600 pixels) and two covering the toys (450 × 600 pixels each; see Fig. 4). GF was assessed using the first look paradigm, where the infant’s initial gaze shift from the actor to either toy was recorded as either congruent or incongruent. GF was calculated by subtracting the number of incongruent trials from the number of congruent trials, resulting in a difference score. Over six trials, this score range from − 6 to 6, with a positive score indicating greater GF. A trial was classified as valid if the child looked at the person and then at one object (regardless of whether it was the same object as the actor looked at or not). At least two valid trials were necessary to be included in further analyses. No participants were excluded due to this criterion. In total, 50 participants were included in the GF analysis. The number of invalid trials (where the infant did not look at any of the toys) are presented separately for each ethnicity in Supplementary Information S3.

### Statistical analyses

To test *H1*, we first performed a Pearson’s correlation in order to assess the association between the EMI when viewing the original videos versus the AI-manipulated videos. Then, we calculated repeated measures ANOVAs with age and sex as covariates, separately for the RunwayML videos and the DeepFaceLab videos. Finally, we performed Bayesian repeated measures analyses.

In relation to *H2*, due to confounding factors (e.g., different ethnicities and acting among the AI-manipulated videos), we only aimed to test whether the children followed gaze in an expected manner. This was done using a one-sample t-test against a chance level of 0.

## Results

Descriptive statistics of all eye tracking measures are shown in Table 2 (see Supplementary Figure S1 for distributional plots of the EMI variable). The EMI was not associated with looking time at the screen when viewing original videos (*r* = − 0.13, *p* = 0.401), RunwayML AI videos (*r* = − 0.25, *p* = 0.098), or DeepFaceLab AI videos (*r* = 0.20, *p* = 0.282). In addition, the EMI was not associated with looking time at the face when viewing original videos (*r* = − 0.05, *p* = 0.735), RunwayML AI videos (*r* = − 0.26, *p* = 0.078), or DeepFaceLab AI videos (*r* = 0.04, *p* = 0.849).

**Table 2:** Descriptive statistics of eye tracking measures

	Mean (SD) [Min; Max]		
	Original videos	AI DeepFaceLab	AI RunwayML
*EMI*			
Looking time at screen (seconds)	9.13 (1.34) [3.71; 9.98]	9.45 (0.07) [6.77; 10.0]	9.38 (1.27) [3.95; 10.0]
Looking time at face (seconds)	8.70 (1.42) [3.26; 9.89]	9.10 (0.08) [6.68; 10.0]	8.91 (1.33) [3.47; 10.0]
Eye-mouth-index	0.54 (0.28) [0.00; 1.00]	0.52 (0.29) [0.05; 1.00]	0.60 (0.27) [0.01; 1.00]
*GF*			
GF difference score	–	–	2.56 (1.93) [− 1; 6]
N valid trials	–	–	4.60 (1.25) [2; 6]

*EMI* eye-mouth-index, *GF* gaze following.

**Fig. 5:**
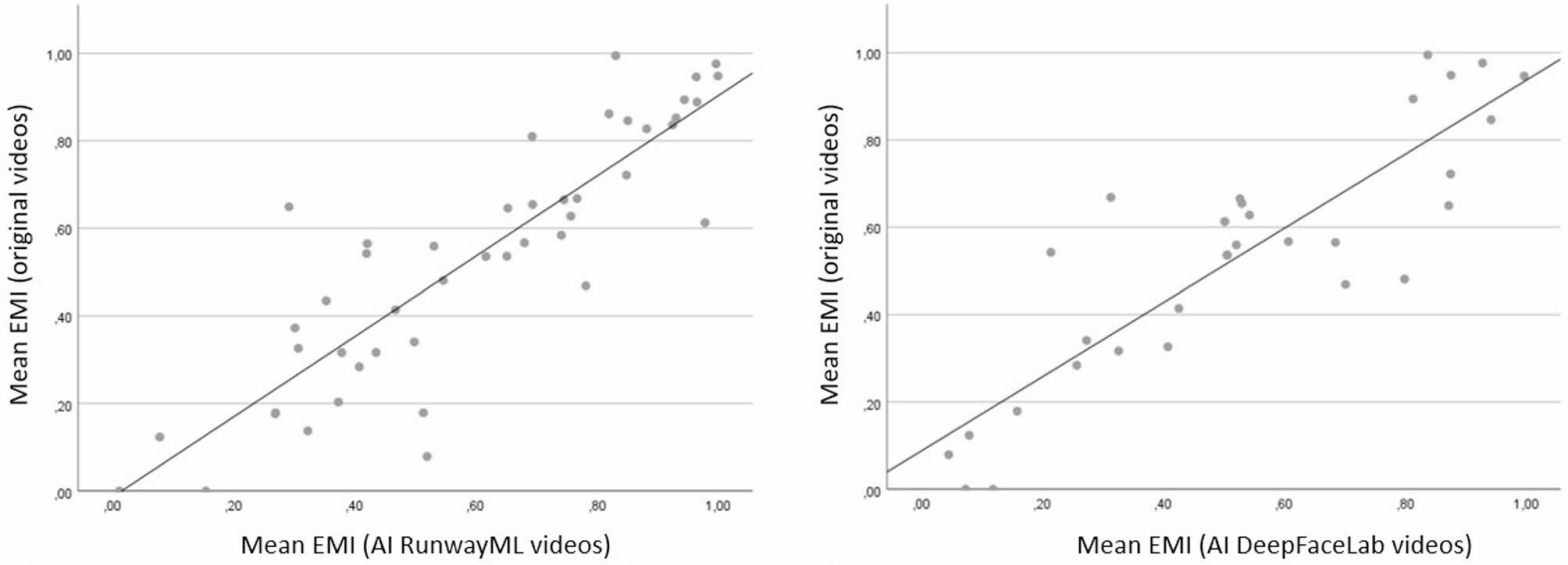
Scatterplot of the correlation between EMI scores for the AI videos (x axis) and the original videos (y axis)

There was a very strong and statistically significant correlation between the EMI in the original condition and the EMI in the RunwayML AI condition (*r* = 0.873, *p* < 0.001; Fig. 5). Similarly, the correlation between the EMI in the DeepFaceLab AI condition and the EMI in the original condition was very strong and statistically significant (*r* = 0.874, *p* < 0.001). These correlations were very similar to the results obtained when comparing actor A to actor B (with EMI values collapsed over the original and RunwayML conditions; *r* = 0.869, *p* < 0.001).

On a group level, the mean EMI was slightly higher when viewing the RunwayML AI videos (mean = 0.60) than when viewing the original videos (mean = 0.54), but this difference was not statistically significant (F(1,43) = 2.517, *p* = 0.120). However, the BF_10_ of this analysis was 6.52, providing some support for the alternative hypothesis (i.e., the alternative hypothesis being approximately 6.52 times as likely as the null hypothesis). No significant effect was found of age (F(1,43) = 0.001, *p* = 0.982) or sex (F(1,43) = 1.452, *p* = 0.235). There was a slight difference in mean EMI in the DeepFaceLab AI condition (mean = 0.52) and in the original condition (mean = 0.54), which was not statistically significant (F(1,28) = 0.236, *p* = 0.631). The BF_10_ of this analysis was 0.20, providing some support for the null hypothesis (i.e., the null hypothesis being approximately 4.95 times as likely as the alternative hypothesis). No significant effect was found of age (F(1,28) = 0.230, *p* = 0.635) or sex (F(1,28) = 0.700, *p* = 0.410).

As additional sensitivity analyses, that were not pre-registered, we analyzed the correlation between the EMI for original and AI videos (RunwayML and DeepFaceLab) separately for videos with person A and person B, as well as analyzing potential differences in the mean EMI. The pattern of results remained largely the same (see Supplementary Information S4 for full details).

**Fig. 6:**
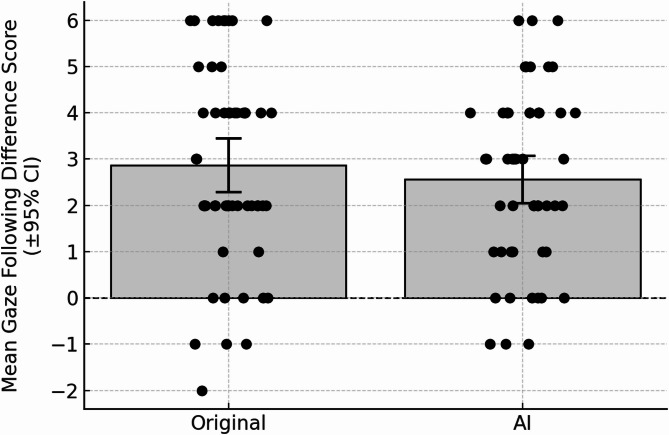
Mean gaze‑following difference scores for the original and AI‑manipulated conditions (*N* = 50). Bars show the group means, with T‑caps indicating the 95% confidence intervals. Individual infant scores are overlaid as jittered, filled black circles. The dashed horizontal line at zero denotes no gaze‑following difference; positive values indicate a greater tendency to follow the actor’s gaze toward the target object

In the GF videos, the children followed the gaze as expected (Fig. 6), as the difference score was significantly different from zero (mean = 2.56, t(49) = 9.38, *p* < 0.001, Cohen’s d = 1.33). The difference score was not related to number of valid trials (*r* = 0.265, *p* = 0.063).

While the primary aim of the GF stimuli was not a comparison to non-AI videos, we added an un-registered analysis, prompted by a reviewer, where we compared the difference score for the AI GF videos to the difference score for non-AI videos, using a repeated measures ANOVA with age and sex as covariates. The non-AI GF videos (6 in total) were shown to this sample during the same eye tracking experiment as the AI stimuli (as part of another study), but they differed from the AI GF videos with regards to the spatial disposition of the scene, the length of the stimuli presentation, the greeting phase, the emotional expression of the actor, and the toys presented (see Supplementary Information S5 for a full overview of this stimuli and the additional analysis). No significant difference in GF was found between the AI stimuli and the non-AI stimuli (F(1,47) = 2.336, *p* = 0.133). No significant effect was found of age (F(1,47) = 1.930, *p* = 0.171) or sex (F(1,47) = 3.565, *p* = 0.065). In addition, we added sensitivity analyses where we compared the difference scores among the three ethnicities in the AI stimuli, and found no significant difference between any of them (see Supplementary Information S5).

## Discussion

The main aim of this study was to assess the feasibility of using AI to create diverse stimuli by examining whether infants demonstrate similar gaze patterns when viewing AI-manipulated videos as when they view non-AI original videos. We focused on two well-known paradigms in infant research: the eye-mouth-index (EMI) and gaze following (GF). Overall, we found no meaningful differences in gaze patterns between original and AI-manipulated videos.

The tendency to look at eyes versus mouth was similar across videos, although the EMI was slightly higher when viewing AI videos generated with the RunwayML software (mean = 0.60) as compared to original videos (mean = 0.54) or AI videos generated with the DeepFaceLab software (mean = 0.52). In other words, there was a group-level tendency to look slightly more at the eyes regardless of video stimuli, but this tendency was more pronounced when viewing the RunwayML AI material. While the difference in mean EMI between original versus RunwayML videos was not statistically significant, the Bayesian analysis suggested some support for the alternative hypothesis. This might be the result of higher contrasts in the AI videos (we changed the eye color from brown in the original videos to blue in the AI-manipulated videos), as well as more prominent facial features and expressions. However, the correlation between the EMI in original versus RunwayML videos was very strong (*r* = 0.87). Notably, that correlation was even stronger than the correlation between the two original videos (*r* = 0.79), and similar to the correlation between actor A and actor B when the EMI measure was collapsed across the original and RunwayML conditions (*r* = 0.87). This suggests that any potential difference in infant performance between original and AI-manipulated stimuli does not exceed the natural variation in infant responses to different actors across original videos. However, when using AI-manipulation, one should carefully examine the stimuli for obvious undesired differences, for example in contrast and lighting—just as one would when creating stimuli through traditional methods. Possibly, the best approach to ensure consistency is to use AI-manipulated stimuli across all study sites (as opposed to comparing an original video to AI-manipulated videos at other sites).

The DeepFaceLab stimuli provided a more direct comparison to the original videos, as they preserved the facial features and lighting of the original videos. The mean EMI for the DeepFaceLab videos was very similar to the EMI in the original videos, and the Bayesian analysis supported the null hypothesis of there being no difference between the means. This further underscores that AI-tools can be successfully utilized in studies of the EMI.

There was a strong and significant tendency to follow gaze when viewing AI-manipulated videos (mean difference score = 2.56), suggesting that these videos can be successfully used to study GF. The mean difference score in our sample compares well with other studies of infant GF, using original videos of actors and the same number of trials as the current study (e.g.,^4,27^). However, these studies include younger infants, and since developmental changes in GF throughout infancy are well documented (e.g.,^3,7,28^) we also compared GF when viewing AI-manipulated videos to GF when viewing non-AI videos in the same eye tracking session, and found no significant differences. While these stimuli were not originally meant to be compared and therefore differed with regards to, for example, the spatial disposition and the greeting phase, this result highlights the stability of the GF behavior in the AI condition.

These findings indicate that infants view AI-manipulated material in largely the same way as original videos, suggesting that AI technology is a promising tool for future studies on social attention. By utilizing available software, we can create diverse stimuli that are perfectly matched in relation to sound and movement, thereby minimizing confounding effects associated with individual differences in performance. In addition, this method creates new opportunities for the global expansion of psychological research, which is crucial for assessing the generalizability of existing findings across non-WEIRD populations^19^.

In this study, we examined two different AI software for creating stimuli for two different social attention paradigms. While the EMI videos included the complexity of a moving mouth, the GF videos included a head turn and static objects in relation to the person in the video. Considering the intricacy of the scenes, we perceived the RunwayML videos as very realistic and without artifacts, even in more complex scenes. While the DeepFaceLab software allowed us to create stimuli that closely matched the visual appearance of the original video and offered a higher degree of control, the process was time-consuming, complex, and less flexible for use in more dynamic scenes. As a result, this software is less suitable for videos including head turns, such as the GF stimuli. Additionally, it is noteworthy that the RunwayML videos were produced within minutes, without requiring specific hardware configurations.

## Limitations

It is important to note that we only tested infants at a certain age range, and these results may not be generalizable to younger or older children. The question of generalizability is even more intriguing considering our experience that adults, including experienced colleagues, were surprised to learn that the AI videos did not feature real people. This highlights the need for further research across age groups and contexts to understand how AI-manipulated stimuli are perceived and how they can be used in research. Our experience in using these tools suggests that it is possible to create stimuli that are engaging and ‘natural’-looking, convincing not only to infants but also adults. This question should be further assessed.

Another limitation is that we only assessed two different paradigms in this study. Future research is needed to determine the boundaries of these methods, perhaps not only in creating stimulus packages with variations but also in refining research stimuli to appear more ‘natural’ and expressive. Implementing AI in the stimuli creation process could make the process more efficient while enabling greater post-production control, such as precise timing adjustments, without introducing artifacts. This could improve the stimulus creation process far beyond its highlighted application in cross-cultural research.

## Conclusions

In this study, we assessed the feasibility of using AI-manipulated material to examine infant gaze patterns. We found that the tendency to look at eyes versus mouth was very similar when viewing original videos and AI-manipulated videos, with a notably high correlation across conditions. In addition, we found that infants follow the gaze of individuals in AI-manipulated videos in a manner consistent with previous literature and at levels comparable to those observed with non-AI videos. In conclusion, AI technology can help us create ecologically valid and culturally diverse stimuli, which can be used to expand developmental research.

## Electronic supplementary material

Below is the link to the electronic supplementary material.

Supplementary Material 1

## Data Availability

The analyses presented here were preregistered (https://osf.io/6vfrd/), and all videos are available at the same link. The data and code necessary to reproduce the analyses presented here are not publicly accessible, but will be made available upon reasonable request to the corresponding author. Note that sharing of pseudonymized personal data will require a data sharing agreement, according to Swedish and EU law.
