# Supplementary material for: A novel approach to exploring infant gaze patterns with AI-manipulated videos
Source: Sci Rep. 2025 Jun 20;15:20097. doi: 10.1038/s41598-025-02727-z (PMC12181369; doi:10.1038/s41598-025-02727-z)
Supplement: Supplementary file 1 — Supplementary Material 1 [file 41598_2025_2727_MOESM1_ESM.docx]

**Supplementary Information**

A Novel Approach to Exploring Infant Gaze Patterns with AI-Manipulated Videos

Charlotte Viktorsson^1*^, Tobias Lundman^1^, & Kim Astor^2^

^1^Development and Neurodiversity Lab, Department of Psychology, Uppsala University; Uppsala, Sweden

^2^Uppsala Child and Baby Lab, Department of Psychology, Uppsala University, Uppsala, Sweden

*Corresponding author. Email: charlotte.viktorsson@psyk.uu.se

**Supplementary Information S1**. Text prompts for the videos generated in RunwayML.

The text prompt for the video in **Figure 1c** was “A young friendly-looking Finnish woman is looking at the camera while singing nursery rhymes. No makeup. Blonde hair and blue eyes. Low contrast and low reflections. Light grey monochrome background.” and the prompt for **Figure 1d** was “A young friendly-looking Finnish woman is looking at the camera while singing nursery rhymes. No makeup. Blonde hair and blue eyes. The woman is shown in color with low contrast and low reflections. The background is monochrome light grey.”

The prompt for **Figure 2b** was “Friendly looking Asian man smiles and says hi and then looks at one of the objects on the table in front of him.”, the prompt for **Figure 2c** was “Friendly looking African woman smiles and says hi and then looks at one of the objects on the table in front of her.”, and the prompt of **Figure 2d** was “Friendly-looking European blonde woman smiling and saying hi before looking at one of the objects in front of her. The background is bright as her hair.”

**Supplementary Information S2**. DeepFaceLab stimuli.

DeepFaceLab (DFL) is an open source software for face swapping (Perov et al., 2020). Although DFL is primarily developed for the swapping of faces, it also has the ability to do a full head swap, which is what was used in the present study.

DFL falls under one-to-one face-swapping paradigm, which means that there are only two types of data; source data and destination data. We have a source video with a face (or head) that we want to transfer on to a destination video. DFL works based on a single pipeline that can be divided into 3 separate phases: 1) extraction, 2) training, and 3) conversion (Perov et al., 2020). Detailed information about these phases are outlined below.

1. Extraction

The extraction phase consists of face detection, face alignment, and face segmentation processes. In short, the DFL program scans all frames of the video, finding the face in each frame using the S3FD algorithm. After it finds a face, it uses facial landmark algorithms (2DFAN or PRNet) to mark out specific parts of the face. Then, each frame is aligned against a template “standard” face that is incorporated in the software. This process makes sure that major features of the face, like nose and eye position, are consistent across each frame. It also makes sure that the face is aligned to a standard orientation, and they are all resized to the same scale. During face segmentation a mask is created of the relevant area of the face. DFL has an automatic face segmentation (TernausNet) but also provides the option for the user to create their own mask by hand. The provided mask is done for faces, while if you want a full head, you must create your own mask using XSeg. This process is done in the same way for both the destination video and the source video. This phase ends with having two folders with aligned faces from both the source and destination video, along with corresponding masks for each frame. This makes us ready to begin training the model.

2. Training

The model used for the training phase was the Sparse Auto Encoder HD (SAEHD), which utilizes a neural network architecture to discover structure within unlabeled data to develop a compressed representation of the input (see https://www.jeremyjordan.me/autoencoders/ for a description).

To train the model, DFL uses a mixed loss by default, using a combination of Structural Dissimilarity Index (DSSIM) and Mean Squared Error (MSE) functions. A loss function measures how well a model’s output matches the expected result. DSSIM is essentially a reverse of Structural Similarity (SSIM). SSIM measures how structurally similar an image is based on pixel-by-pixel comparison (Wang et al., 2004). DSSIM does the same but looks for dissimilarity instead. DSSIM is good at generalizing human faces fast, while MSE improves the clarity (Perov et al., 2020). By using the mixed loss functions described above, the model can compare the input face it receives, both destination and source face, to the output that it produces, and over time improve the similarity between the input and its output. This is the actual training of the model, that over time gets better at encoding features of the source face and can then map these features to the destination face using the destination decoder.

3. Conversion

Conversion is the last phase of the face swap. Here, the generated face is transformed with its mask to the original position of the target image in the source video (Perov et al., 2020). DFL provides five color transformation algorithms to help approximate the color of the constructed face to the target image, as well as blending to account for differences in skin tones, illumination or face shapes.


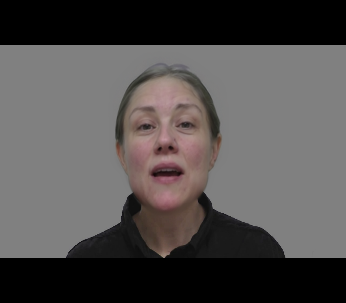

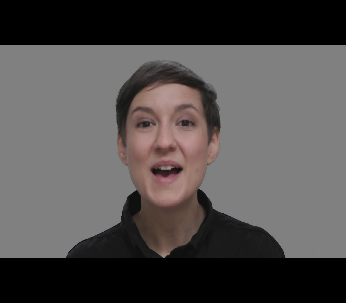

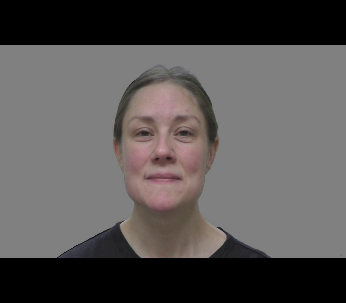
Actor A


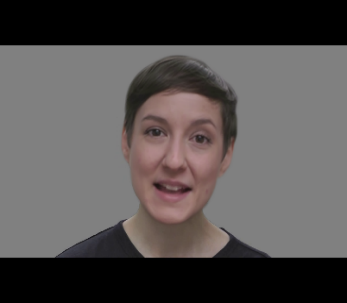

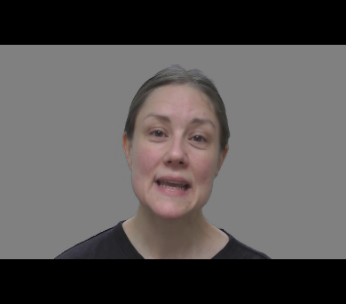

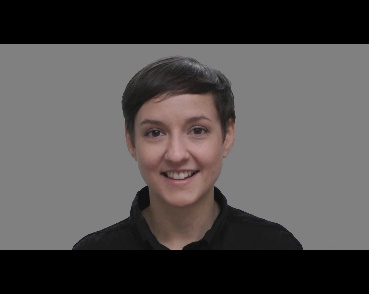
Actor B

The figures above show a simplified overview of the process and the materials used. To the left is the source video of actor A and B, in the middle is the destination video for each actor (i.e., the original video of the other actor), and to the right is the resulting video where the head from the source video has been placed on the destination video.

The training process for actor A required 702,123 iterations on a Windows 10 system with an Intel Core i5-8600K and 16 GB of RAM and a NVIDIA GeForce GTX 1080 Ti GPU, while the training process for actor B required 254,611 iterations (a difference that was mainly due to time restraints) on a Windows 11 system with an AMD Ryzen 5 7600X and 32 GB of RAM and an NVIDIA GeForce RTX 4070 Super. The difference in hardware forced us to use two different versions of the DFL software; *DeepFaceLab_NVIDIA_up_to_RTX2080TI_Build_11_20_2021* for the manipulation of actor A and *DeepFaceLab_NVIDIA_RTX3000_series_build_11_20_2021* for the manipulation of actor B (available here; <https://mega.nz/folder/Po0nGQrA#dbbttiNWojCt8jzD4xYaPw>).

Additionally, a pretrained model was used to enhance the training of both actors: DF-UD HEAD 320 SAEHD pretrained model. This model has been pre-trained for 1 million iterations on heads and is available for download at <https://www.deepfakevfx.com/pretrained-models-saehd/>. Using pre-trained models greatly reduces the time for the AI model to find patterns and thus reduces the time of training.

For a more in-depth account of the workflow involved in creating a DFL video see; <https://www.deepfakevfx.com/guides/deepfacelab-2-0-guide/>.

**Supplementary Information S3.** Invalid trials across ethnicity.

While the aim of this study was not to investigate differences between stimuli where different ethnicities are presented, we did examine the number of invalid trials (where the infant did not look at any of the toys) separately for each ethnicity. There was a similar number of non-response trials for the Scandinavian female and the African female (18 and 16, respectively, added together for all trials and all participants). However, there were more non-responsive trials for the Asian male (30, in total).

**Supplementary Information S4.** Sensitivity analyses.

As additional sensitivity analyses, that were not pre-registered, we analyzed the correlation between the EMI for original and AI videos (RunwayML and DeepFaceLab) separately for videos with person A and person B, as well as analyzing potential differences in the mean EMI. The pattern of results remained largely the same, namely that the correlations between original and AI videos were high and statistically significant for both the RunwayML videos (person A: 0.751, p <.001; person B: r = 0.866, p <.001) and for the DeepFaceLab videos (person A: r = 0.804, p <.001; person B: r = 0.593, p <.001). After controlling for age and sex, the mean differences between original and AI videos were not statistically significant for the RunwayML videos (person A: F(1,43) = 2.530, p = 0.119; person B: F(1,43) = 0.596, p = 0.444) or the DeepFaceLab video for person B (F(1,28) = 1.204, p = 0.320). The difference was significant for the DeepFaceLab videos of person A (F(1,28) = 4.381, p = 0.046), although the mean values were very similar (mean EMI original = 0.526; mean EMI DeepFaceLab = 0.516).

**Supplementary Information S5.** Comparing AI GF stimuli to non-AI GF videos.

The non-AI GF videos included in the eye-tracking experiment were performed by three different women. Each trial began with the actor facing downward toward the table during an initial 2-second pre-phase. looking at the camera after a “beep” sound, then looking at one of the toys (see figure below). In contrast to the AI GF videos, the women did not smile or say “Hello”, and the toys were slightly different. The length of the stimuli differed, with longer ones for the non-AI stimuli, and the spatial layout differed, prompting differences in AOI definition. We never intended to compare the AI GF stimuli and these non-AI GF videos (that were part of another study). Nevertheless, the mean difference score for the original videos (mean = 2.88) was comparable to the difference score for the AI GF videos (mean 2.56), and a repeated measures ANOVA revealed no significant differences between these videos (F(1,47) = 2.336, p = 0.133). While no difference was found, we added three sensitivity analyses where we compared the difference score for each ethnicity in the AI videos, as there may be potential differences between ethnicities. There were no differences between gaze following for the Scandinavian woman and the African woman (t(21) = 0.224, p = 0.825), or between the Scandinavian woman and the Asian male (t(23) = 1.446, p = 0.162). Similarly, no difference was found between the African woman and the Asian male (t(18) = 0.294, p = 0.772). For each comparison, participants were only included if they had two valid trials for each ethnicity. The figure below was republished from Astor et al. (2020) with permission from authors. However, we were unable to confirm whether written permission to publish identifiable information was obtained from the person in the figure.


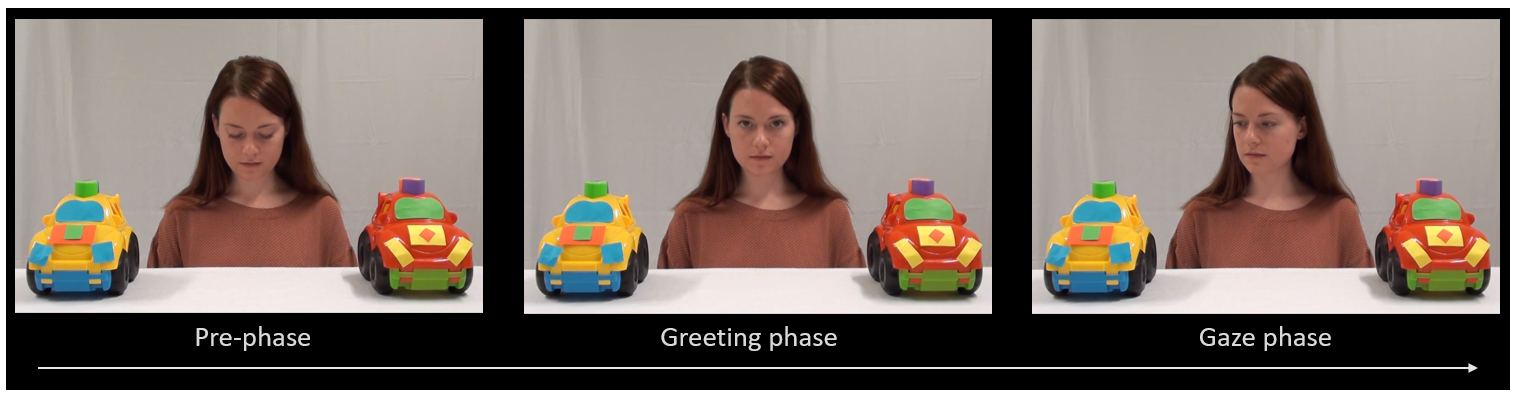


**Tables and Figures**

**Supplementary Figure S1**. Distributional plots of EMI scores in each condition.


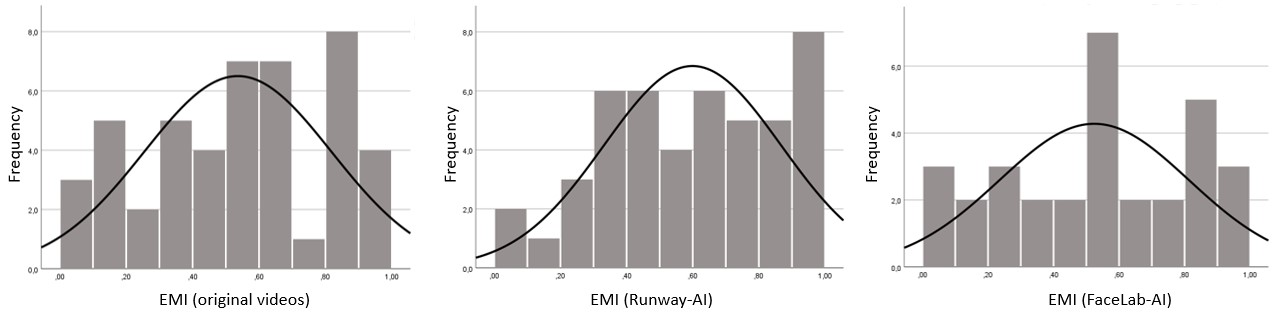


**References**

Astor, K., Lindskog, M., Forssman, L., Kenward, B., Fransson, M., Skalkidou, A., Tharner, A., Cassé, J., & Gredebäck, G. (2020). Social and emotional contexts predict the development of gaze following in early infancy. *R. Soc. Open Sci*. 7201178 <http://doi.org/10.1098/rsos.201178>

Perov, I., Gao, D., Chervoniy, N., Liu, K., Marangonda, S., Umé, C., ... & Zhang, W. (2020). DeepFaceLab: Integrated, flexible and extensible face-swapping framework. *arXiv preprint arXiv:2005.05535*.
